# Draft Genome Sequence of a Multidrug-Resistant and Novel Sequence Type 6130 Klebsiella quasipneumoniae Strain C11S11_BCSIR Isolated from Wastewater of a Tertiary Care Hospital in Chattogram, Bangladesh

**DOI:** 10.1128/mra.00654-22

**Published:** 2022-08-29

**Authors:** Jannatul Ferdous, Saddam Hossain, Farjana Showline Chaity, Rajib Sarkar, Anamul Bahar, H. M. Abdullah Al Masud, Saiful Islam

**Affiliations:** a Industrial Microbiology Research Division, BCSIR Chattogram Laboratories, Bangladesh Council of Scientific and Industrial Research (BCSIR), Chattogram, Bangladesh; b University of London, Senate House, Malet Street, London, United Kingdom; c Department of Microbiology, Faculty of Biological Sciences, University of Chittagong, Chattogram, Bangladesh; Vanderbilt University

## Abstract

In this article, we report draft genome sequence and annotation of Klebsiella quasipneumoniae from the wastewater source in Bangladesh. Here, we identified Klebsiella quasipneumoniae strain C11S11_BCSIR, a multidrug-resistant pathogenic bacterium harboring seven antimicrobial resistance genes of five major antibiotic classes with a novel multilocus sequence type (MLST) (ST6130).

## ANNOUNCEMENT

Klebsiella quasipneumoniae, a phylogroup (Kp2) of Klebsiella pneumoniae (1), is commonly found in the gastrointestinal tracts of humans (2). According to studies, the pathogen can be transmitted from human to environment, resulting in the dissemination of antibiotic resistance genes (3). A chromosomally encoded *blaOKP-B-2* gene is frequently found in Klebsiella quasipneumoniae subsp. *similipneumoniae* (4). Here, we used whole-genome sequencing to describe the genetic characteristics of Klebsiella quasipneumoniae strain C11S11_BCSIR.

Previously, the wastewater sample was collected from a tertiary care hospital (22.36 N 91.83 E) in Chattogram city in 2019. The sample was filtered through a 0.45-μm membrane filter, and subsequently, the filter was transferred into a buffer peptone water medium (Himedia, India) for enrichment at 37°C for 24 h. The enriched culture was then subcultured onto MacConkey agar medium (Himedia, India) and incubated at 37°C for 24 h. The large, mucoid, and pink colonies were identified as Klebsiella quasipneumoniae after extraction of DNA (5) followed by the amplification in a multiplex PCR scheme using the primers OKP-F (5′-GGCCGGYGAGCGGGGCTCA-3′) and DeoR-R (5′-AGAAGCATCCTGCTGTGCG-3′) (6). Following Clinical and Laboratory Standards Institute (CLSI) guidelines (7), an antibiotic susceptibility test was carried out by disc diffusion method employing available antibiotics (Oxoid, United Kingdom).

Genomic DNA was extracted from the overnight culture using the Epicenter MasterPure extraction kit (Epicentre), and then the library was prepared using the Nextera XT library (Illumina, San Diego, USA). Illumina NextSeq 550 with 150 bp paired-end reads was used for sequencing and finally yielded 1,958,888 reads with 54× coverage. FastQC v0.11.9 was used to check the quality of the raw reads, and the adapter sequences were eliminated by Trimmomatic v0.39. The raw reads were assembled by *de novo* assembly using SPAdes v3.14.1. The genome quality had been assessed using QUAST v5.2.0 (8). The NCBI PGAP (Prokaryotic Genome Annotation Pipeline) was used for the annotation of the genome. The Center for Genomic Epidemiology’s (https://www.genomicepidemiology.org/) ResFinder 4.1, multilocus sequence type (MLST) 2.0, and PlasmidFinder 2.0 programs were used to identify antibiotic resistance genes (ARGs), MLST profile, and plasmids, respectively. Kleborate v2.2.0 (9), together with Kaptive v2.0.3 (10), was used to determine the species identity, virulence loci, capsule, and LPS antigen in Klebsiella quasipneumoniae. All applications were used with default parameters except when otherwise noted.

Our strain C11S11_BCSIR was identified to be Klebsiella quasipneumoniae subsp. *similipneumoniae* using Kleborate, which was later validated by the NCBI’s average nucleotide identity of 99.21%. The genome assembly contained 5,431,876 bp, including two circular plasmids (pIncFII-K and pIncFIB-K-pCAV1099-1), 91 contigs, *N*_50_ value of 198,177 bp whereas L50 of 11 bp, GC content of 57.51%, and genome coverage of 86.22%. A total of 5,377 genes were found after analyzing NCBI PGAP output, including 5,133 coding genes, 154 pseudogenes, 3 rRNAs, 76 tRNAs, and 11 noncoding RNAs. The strain was phenotypically multidrug resistant, exhibiting resistance to ampicillin, amoxicillin-clavulanic acid, piperacillin-tazobactam, streptomycin, amikacin, gentamicin, ciprofloxacin, ceftriaxone, and cefotaxime. Several ARGs such as *blaCTX-M-15*, *blaTEM-1B*, *aph(3″)-Ib*, *aph(6)-Id*, *sul2*, *qnrS1*, and *fosA* were identified after resistome analysis. Presence of the *blaOKP-B-2* gene supports the identification of our strain. Chromosomal point mutations were observed in *gyrA, gyrB, parC*, and *acrR* genes encoding resistance to fluoroquinolones. The MLST profile was curated by an MLST database (https://bigsdb.pasteur.fr/klebsiella/), and our strain was assigned for a novel sequence type, ST6130. The strain C11S11_BCSIR was further identified as an O4 serotype with a capsule (KL139).

### Data availability.

The present whole-genome shotgun project has been deposited in DDBJ/ENA/GenBank under accession no. JAMSHK000000000 and BioProject accession no. PRJNA845367. The version described in this paper is version JAMSHK010000000. The raw reads have been deposited in the Sequence Read Archive (SRA) under accession no. SRR19572342.
